# Chronic rapamycin treatment on the nutrient utilization and metabolism of juvenile turbot (*Psetta maxima*)

**DOI:** 10.1038/srep28068

**Published:** 2016-06-16

**Authors:** Qingchao Wang, Gen He, Kangsen Mai, Wei Xu, Huihui Zhou, Xuan Wang, Lin Mei

**Affiliations:** 1 Key Laboratory of Aquaculture Nutrition and Feed, Ministry of Agriculture, Ocean University of China, Qingdao, China; 2 College of Fisheries, Key Lab of Freshwater Animal Breeding, Ministry of Agriculture, Huazhong Agricultural University, Wuhan, China

## Abstract

High dietary protein inclusion is necessary in fish feeds and also represents a major cost in the aquaculture industry, which demands improved dietary conversion into body proteins in fish. In mammals, the target of rapamycin (TOR) is a key nutritionally responsive molecule governing postprandial anabolism. However, its physiological significance in teleosts has not been fully examined. In the present study, we examined the nutritional physiology of turbot after chronic rapamycin inhibition. Our results showed that a 6-week inhibition of TOR using dietary rapamycin inclusion (30 mg/kg diet) reduced growth performance and feed utilization. The rapamycin treatment inhibited TOR signaling and reduced expression of key enzymes in glycolysis, lipogenesis, cholesterol biosynthesis, while increasing the expression of enzymes involved in gluconeogenesis. Furthermore, rapamycin treatment increased intestinal goblet cell number in turbot, while the expressions of Notch and Hes1 were down regulated. It was possible that stimulated goblet cell differentiation by rapamycin was mediated through Notch-Hes1 pathway. Therefore, our results demonstrate the important role of TOR signaling in fish nutritional physiology.

One of the major goals in animal husbandry is to improve feed conversion to body protein accretion, which is directly correlated with protein synthesis in tissues. Compared to mammals, fish are characteristic for their high dietary protein requirement^1^, high feed conversion efficiency^2^, and indeterminate growth. Protein metabolism in fish is critical for anabolism and weight gain^3^, as well as catabolism and energy production^4^,^5^. However, the underlying mechanism for protein metabolic regulation in fish remains largely unexplored.

In mammals, target of rapamycin (TOR) signaling can acts as the key checkpoint to integrate nutritional signals, such as leucine^6^ and glutamate or glutamine^7^,^8^, with anabolism vs catabolism^9^,^10^. Upon postprandial activation, TOR phosphorylates downstream effectors including p70 S6 kinase1 (S6K1) and eukaryotic initiation factor 4E binding protein 1 (4EBP1), which regulate protein translational initiation^11^,^12^. Chronic rapamycin inhibition of TOR signaling in mammals reduces protein synthesis^13^,^14^,^15^,^16^ and growth^17^,^18^,^19^, as well as developmental arrest^20^,^21^. TOR signaling regulates nutrient metabolism profoundly, as inhibition of TOR reduces lipogenesis^22^,^23^, glycolysis^24^ while promoting fatty acid β-oxidation^25^.

It has been demonstrated that TOR signaling is conserved in teleosts^26^. TOR signaling could be regulated by amino acids in rainbow trout hepatocytes *in vitro*^27^. Meanwhile it could also be regulated by dietary protein levels^28^ and sources^29^ in the feeding experiments. Acute intraperitoneal injection of rapamycin in rainbow trout influenced metabolism^30^,^31^. However, the regulatory role of TOR signaling on growth and feed utilization in fish has not been fully explored. In this study, the growth performance and physiology of turbot after chronic rapamycin inhibition was examined.

## Results

### Chronic rapamycin treatment reduced growth performance and feed utilization

As shown in Table 1, after a 6-week rapamycin treatment, turbot survival was not influenced (100%). However, final body weight (FBW), weight gain ratio (WGR) and specific growth rate (SGR) were significantly reduced. Rapamycin treatment also increased feed intake (FI), feed conversion ratio (FCR) and reduced protein efficiency ratio (PER). The apparent digestibility coefficients (ADC) of dry matter and protein were reduced. Hepatopancreas somatic indices (HSI), condition factor (CF), and body composition, including moisture, crude protein, lipid and ash content, were not significantly affected by rapamycin treatment.

### Rapamycin treatment inhibited TOR signaling

**Figure 1:**
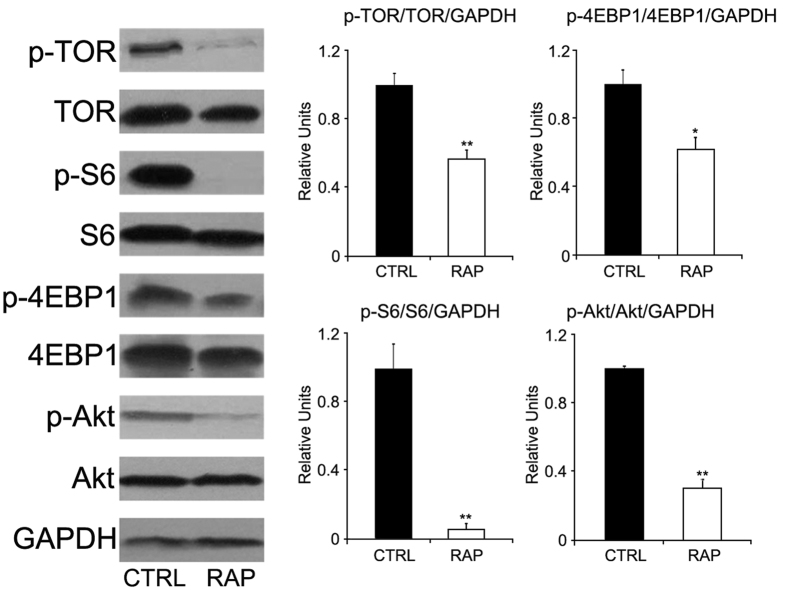
Western blot analysis of TOR signaling after dietary rapamycin treatment. No difference was found in total protein levels of TOR, Akt, 4EBP1 and S6, while their phosphorylation was significantly decreased after rapamycin treatment. Data are means ± SEM (n = 3). The error bars represent standard error of the mean (SEM). Mean values were significantly different from those of the control group: *P < 0.05, **P < 0.01, ***P < 0.001.

The concentration of rapamycin in the blood of fish fed with 30 mg rapamycin/kg diet was 52.34 ± 6.50 ng/ml 3 hrs after feeding. As shown in Fig. 1, No significant differences in total TOR, Akt, S6, and 4EBP1 levels were found between treatments in the liver. However, phosphorylation of TOR, Akt, S6, and 4EBP1 was significantly reduced after rapamycin treatment, suggesting the successful inhibition of TOR signaling.

### Rapamycin treatment modulated nutrient metabolisms

**Figure 2:**
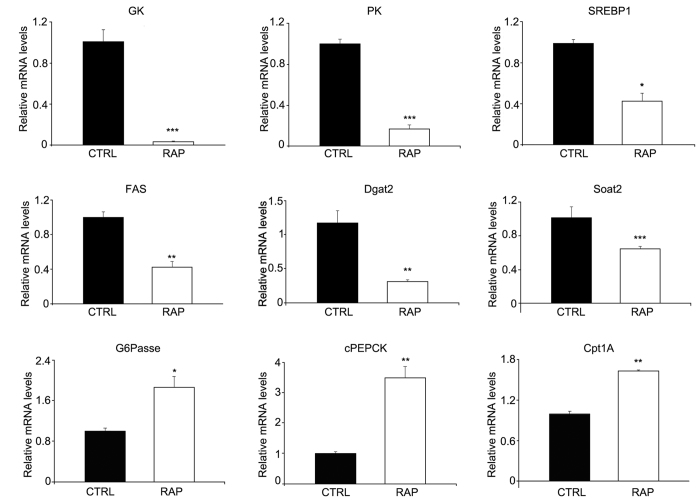
Expression of hepatic metabolic enzymes after rapamycin treatment. Genes involved in glycolysis (glucokinase, GK and pyruvate kinase, PK), fatty acid synthesis (fatty acid synthetase, FAS), triglycerol synthesis (diacylglycerol O-acyltransferase homolog, DGAT2), cholesterol synthesis (sterol O-acyltransferase 2, Soat2) along with regulating factor (sterol regulatory element-binding protein, SREBP1) had reduced expression after rapamycin treatment; while gluconeogenesis (glucose 6 phosphatase, G6Pase; cytosolic phosphoenolpyruvate carboxykinase, cPEPCK) and fatty acid oxidation (carnitine palmitoyltransferase 1 isoforms A, CPT1A) was significantly increased after rapamycin treatment. Data are means ± SEM (n = 6). The error bars represent standard error of the mean (SEM). Mean values were significantly different from those of the control group: *P < 0.05, **P < 0.01, ***P < 0.001.

As shown in Fig. 2, liver expression levels of enzymes for glycolysis (glucokinase, GK and pyruvate kinase, PK), fatty acid synthesis (fatty acid synthetase, FAS), triglycerol synthesis (diacylglycerol O-acyltransferase homolog, DGAT2), cholesterol synthesis (sterol O-acyltransferase 2, Soat2) along with regulating factor (sterol regulatory element-binding protein, SREBP1) were reduced after rapamycin treatment. On the other hand, enzyme expression levels of gluconeogenesis (glucose 6 phosphatase, G6Pase; cytosolic phosphoenolpyruvate carboxykinase, cPEPCK) and fatty acid oxidation (carnitine palmitoyltransferase 1 isoforms A, CPT1A) were upregulated after rapamycin treatment.

### Rapamycin treatment modulated intestinal differentiation

**Figure 3:**
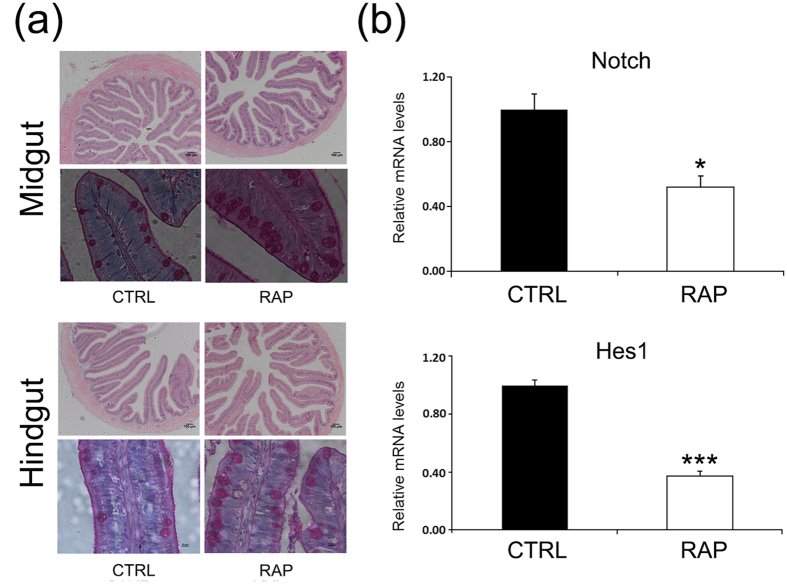
Rapamycin treatment influenced intestinal differentiation. (**a**) H&E and PAS staining of midgut and hindgut of turbot showed rapamycin treatment (RAP) increased numbers of goblet cells. (**b**) Rapamycin treatment reduced expression levels of Notch and Hes1(hairy/enhancer of split 1). Data are means ± SEM (n = 6). The error bars represent standard error of the mean (SEM). Mean values were significantly different from those of the control group: *P < 0.05, **P < 0.01, ***P < 0.001.

The morphology of intestine after rapamycin treatment was examined by both H&E and PAS staining. As shown in Fig. 3, goblet cell number was stimulated after rapamycin treatment. The expression levels of both Notch1 and Hes1, the key signaling molecules involved in intestinal cell differentiation, were down-regulated after rapamycin treatment.

## Discussion

In the present study, rapamycin was introduced into the diet at 30 mg/kg diet. Consequently, rapamycin concentration in the blood was 52.34 ± 6.50 ng/ml 3 hrs after feeding. At this concentration, the postprandial activation of TOR signaling in liver, the most typical and active metabolic organ in fish, was inhibited, which was represented by the phosphorylation of related molecules including TOR, Akt, S6K, 4EBP1. It has been well demonstrated that TOR signaling controls cell growth and protein synthesis in mammals^9^,^16^,^17^. Acute rapamycin treatment led to multiple metabolic changes in rainbow trout^31^,^32^. However, the phenotypical changes in teleost after chronic treatment has not been characterized. In this study, chronic rapamycin treatment significantly reduced the growth performance (such as FBW, SGR and WGR) and apparent digestibility coefficients of dry diet and protein in turbot. These results demonstrated the critical roles of TOR signaling in protein synthesis and growth in teleost.

Meanwhile, chronic rapamycin treatment reduced mRNA expression of enzymes for glycolysis (glucokinase, GK and pyruvate kinase, PK), fatty acid synthesis (fatty acid synthetase, FAS), triglycerol synthesis (diacylglycerol O-acyltransferase homolog, DGAT2), and cholesterol synthesis (sterol O-acyltransferase 2, Soat2). On the other hand, enzyme expression levels of gluconeogenesis (glucose 6 phosphatase, G6Pase; cytosolic phosphoenolpyruvate carboxykinase, cPEPCK) and fatty acid oxidation (carnitine palmitoyltransferase 1 isoforms A, CPT1A) were upregulated after rapamycin treatment. Our results are consistent with previous results obtained in mammals^33^,^34^. Dai *et al*.^30^ showed that acute rapamycin treatment did not influence expression of genes involved in glycolysis and fatty acid oxidation, but decreased those in lipogenesis (SREBP1c, FAS). Together with the results obtained in mammals, it seems that the effects of rapamycin treatment on glycolysis and fatty acid oxidation can only be achieved through chronic treatment. On the other hand, Dai *et al*. showed no influence^30^ but later an inhibitory effect on gluconeogenesis (G6Pase, and FBPase)^31^ after acute rapamycin treatment. Our results showed a stimulatory effect on gluconeogenesis after chronic rapamycin treatment, a result consistent with what has been found in mammals^17^.

Moreover, apparent digestibility coefficients of dry diet and protein were decreased after rapamycin treatment, suggesting an impact on intestinal digestion/absorption. Intestinal epithelium is undergoing continuous self-renewal to maintain function^35^ through stem cell differentiation into cells including nutrient absorbing enterocytes, mucous secreting goblet cells, and enteroendocrine cells that release gastrointestinal hormones^36^.Our result demonstrated that rapamycin treatment increased intestinal goblet cell number in turbot. This represented the first evidence that TOR signaling was involved in intestinal differentiation in teleost. In mammals, it is known that intestinal differentiation is controlled by Notch-Hes1 pathway, inhibition of which stimulated goblet cell differentiation^37^,^38^,^39^,^40^,^41^. Our result further demonstrated that the expressions of Notch and Hes1 were down regulated after rapamycin treatment. This was consistent with recent studies showing that TOR signaling regulated intestinal homeostasis through Notch expression and activities in mice^42^. Taking all these evidences together, it was possible that stimulated goblet cell differentiation by rapamycin in turbot was mediated through Notch-Hes1 pathway.

In summary, the present study demonstrated that TOR signaling played an important role in fish growth, nutrient metabolism and intestinal cell differentiation. Well regulated TOR signaling appears crucial for animal growth homeostasis. TOR signaling is responsive to dietary protein levels^28^ and sources^29^. Meanwhile, it is required for postprandial anabolism. Insufficient postprandial TOR activation would likely result in decreased fish growth and feed efficiency^43^. A better understanding and manipulation of TOR signaling are warranted for optimal dietary design and utilization in aquaculture.

## Materials and Methods

### Ethics statement

The present study was performed in strict accordance with the Standard Operation Procedures (SOPs) of the Guide for the Use of Experimental Animals of Ocean University of China. All animal care and use procedures were approved by the Institutional Animal Care and Use Committee of Ocean University of China. Fish were anesthetized with eugenol (1:10,000) (Shanghai Reagent Corp., Shanghai, China) to mini- mize suffering before being assigned to cages and sampling.

### Feed Ingredients and diet formulation

The basal diet (Table 2) used fishmeal as the main protein source and fish oil as the main lipid source. This basal diet had a protein content of ~50% and lipid content of 12.5% to meet the nutritional requirements of turbot^44^,^45^. All ingredients were ground into fine powder through a 180-μm mesh. The ingredients were then thoroughly mixed with fish oil. For the experimental diet, 30 mg rapamycin (Biomol, PA) per kg diet was dissolved in 0.8 ml ethanol, serially diluted with water and mixed thoroughly with the other ingredients during diet preparation. The control diet was made by introducing the vehicle (0.8 ml ethanol/kg diet) into the ingredient mixture during preparation. Stiff dough was produced and pelletized using a small-scale feed mill (F-26(II), South China University of Technology, China). All diets were stored at −20 °C until used. No differences in physical properties were observed between diets.

### Fish rearing and sampling

Fish rearing and sampling were carried out according to the experimental procedures of the key laboratory of mariculture (Ministry of Education of China), Ocean University of China. Juvenile turbot were purchased from a fish farm in Jiaonan, Shandong, China. The experiment was carried out at the National Oceanographic Center, Qingdao, China. During the acclimatization period, fish were fed a commercial diet (Great Seven Biotech, Shandong, China) twice per day for two weeks.

At the beginning of the experiment, juvenile turbot (19.20 ± 0.10 g) were randomly distributed into tanks filled with 500 liters of seawater with 40 fish in each. Diets were randomly allocated in triplicate among the tanks. Fish were fed twice per day until apparent satiation for 6 weeks. All tanks were connected to a flowing water system with flow rate of 0.5 L/min. Oxygen concentration was maintained at over 85% saturation throughout the study. Day length followed natural changes over trial. Water temperatures in tanks were maintained at 18 ± 1 °C.

At the end of the 6-week rearing experiment, 4 fish were randomly chosen per tank 3 hrs after the final feeding, anaesthetized and sacrificed by a hit on head for blood and tissue analysis using western blots and qRT-PCR. Another 10 fish per tank were randomly chosen 24 hr after the final feeding for body morphometrics, intestinal histology and body composition analysis.

### Biochemical analyses

Proximate composition analyses of diets and fish were performed as described before^43^. Specimens for whole body analysis were dried (105 °C) to estimate water content. Whole blood samples were extracted from the caudal vein for rapamycin analysis. Liver and intestine samples were immediately dissected and frozen in liquid nitrogen for analysis.

Rapamycin was assayed as described before^46^. Briefly, zinc sulfate (50 g/L) and acetone were added to blood samples and centrifuged at 2,600 g for 5 min. The supernatant was transferred, then 100 mM NaOH and 1-chlorobutane were added and centrifuged again at 2,600 g for 5 min. The supernatant was then transferred and dried under nitrogen and reconstituted with mobile phase (600 mM acetonitrile, 150 μL). Hexane (500 μL) was added to each sample, mixed, and centrifuged at 2,600 g for 2 min. The hexane layer was removed from each sample and discarded while the extract was transferred to sampler vials. The samples were then injected into an HPLC system with Agela C18 analytical column (Agilent Technologies). The mobile phase was pumped at 0.5 mL/min and rapamycin was detected at 278 nm with a retention time of ~9 min.

### Digestibility

For digestibility assays, faeces were drawn from the bottom of the glass fiber rearing tanks 3 hours after the feeding. The pooled fecal samples were freeze-dried. Yttrium oxide content in the diet and feces were determined according to the method of Mai and Tan^47^. The apparent digestibility coefficients of nutrients were calculated as follows: ADC(100%) = (1−(Y % in diet)/(Y % in faeces)) * (%nutrient or energy in faeces/% nutrient or energy in diet) * (100%). For dry matter, the apparent digestibility coefficients were: ADC (100%) = (1−(Y% in diet)/(Y% in faeces)) * 100%.

### Intestinal histology analysis

Fish intestines were fixed with Bouin’s solution and transferred into 70% ethanol after 24 hrs. Segments of intestine were sliced transversely into 6-μm sections and stained with hematoxylin-eosin (H&E) and PAS staining separately and examined using a light microscope (Olympus, DP72) equipped with camera (Nikon E600). Images were acquired using CellSens Standard Software (Olympus).

### RNA extraction and quantitative PCR analysis

Total RNA samples were extracted using TRIZOL reagent (Invitrogen, Carlsbad, CA, USA) according to the manufacturer’s instructions. Integrity of RNA samples were tested by electrophoresing on a 1.2% denaturing agarose gel while the purity and concentration were determined by NanoDrop ND-1000. The absorption ratios (260:280 nm) for all of the samples were approximately 2.00. One microgram of total RNA was reverse transcribed into cDNA using the SuperScript III RNaseH-Reverse Transcriptase kit (Invitrogen, Carlsbad, CA, USA) according to the manufacturers’ instructions.

Primers for metabolic genes were synthesized according to Xu *et al*.^29^ and Cunha *et al*.^48^. Core sequences of Notch and Hes1 in turbot were attained as described by Yuan *et al*.^49^ and primers were then designed using Primer 5 software. All primers used in the present study are listed in Table 3. Targeted gene expression levels were determined by quantitative RT-PCR carried out on an iCycler iQTM real-time PCR detection system (BIO-RAD, Hercules, CA, USA) using iQ TM SYBR Green Supermix. The thermal cycle program was as follows: 95 °C for 2 min, followed by 40 cycles of 95 °C for 10 s, 58 °C for 10 s, and 72 °C for 20 s. At the end of each PCR, melting curve analysis was performed to confirm that only one PCR product was present in these reactions. Data were expressed as the ratio between the expression of the target gene and the housekeeping genes EF1α (for liver) and α-tubulin (for intestine), the expression of which showed similar efficiencies like target genes and were not significantly affected by treatment (data not shown). To calculate relative expression levels, the comparative CT method (2^−ΔΔCt^ method) was used^50^.

### Western blot analysis

Tissues were homogenized in RIPA buffer (20 mM Tris, pH 7.5, 140 mM NaCl, 1% Nonidet P-40, 10 mM NaF, 2 mM Na_3_VO_4_). Proteins were separated by SDS-PAGE and transferred to PVDF membrane (Pall Corporation). Anti-TOR (cat # 2972), anti-phospho-TOR (Ser2448) (cat # 2971), anti-Akt (cat # 9272), anti-phospho-Akt (Ser473) (cat # 9271), anti-S6 (cat # 2217), anti-phospho-S6 (Ser235/236) (cat # 4856) , anti-4E-BP1 (cat # 9452), anti-phospho-4E-BP1 (Thr37/46) (cat # 9271) were purchased from Cell Signaling Inc. USA. Anti-GAPDH (AB-P-R 01) was purchased from Hangzhou Goodhere Biotechnology, China.

### Calculations and statistical analysis

Growth parameters were calculated as follows:

Specific Growth Rate (%/day) = 100 * (Ln W_t_ − Ln W_0_)/t;

Weight Gain Ratio (%) = (W_t_ − W_0_)/W_0_;

Feed Intake (%/day) = 100 × dry feed intake × 2/(W_t_ + W_0_) × t;

Protein Efficiency Ratio = (W_t_ − W_0_)/(dry feed intake * protein percent in dry diet);

Feed Conversion Ratio = (dry feed intake)/(W_t_ − W_0_);

Hepatopancreas Somatic Indices (%) = 100 * (liver weight/whole body weight);

Condition Factor (%) = 100 * (body weight/body length^3^).

W_t_ and W_0_ represented the body weight of initial fish and final fish respectively, while *t* represented the rearing days of this experiment.

### Statistical analysis

Statistical analysis was performed by using SPSS 16.0 (SPSS Company, Quarry Bay, Hong Kong). Student’s t-tests were performed to compare differences between two groups. Data are presented as means ± SEM. The error bars represent standard error of the mean (SEM).

## Additional Information

**How to cite this article**: Wang, Q. *et al*. Chronic rapamycin treatment on the nutrient utilization and metabolism of juvenile turbot (*Psetta maxima*). *Sci. Rep.* **6**, 28068; doi: 10.1038/srep28068 (2016).

## Figures and Tables

**Table 1:** Growth performance and body features of turbot after rapamycin treatment.

	**CTRL**	**RAP**	***P*** **value**
Final body weight (FBW, g)	52.87 ± 0.12	44.67 ± 0.26	0.000
Specific growth rate (SGR, %/day)^1^	2.41 ± 0.01	1.98 ± 0.01	0.000
Weight gain ratio (WGR)^2^	1.76 ± 0.01	1.30 ± 0.01	0.000
Feed intake (FI, %/day)^3^	1.53 ± 0.00	2.18 ± 0.02	0.000
Hepatopancreas Somatic index (HSI, %)^4^	1.04 ± 0.21	1.06 ± 0.11	0.592
Condition factor (CF, %)^5^	3.79 ± 0.14	4.14 ± 0.50	0.221
Feed conversion ratio (FCR)^6^	0.69 ± 0.00	1.16 ± 0.01	0.000
Protein efficiency ratio (PER)^7^	2.89 ± 0.00	1.72 ± 0.02	0.000
Apparent digestibility coefficient (ADC, dry weight, %)^8^	55.66 ± 1.40	47.40 ± 2.06	0.018
Apparent digestibility coefficient (ADC, protein, %)^9^	87.26 ± 0.41	81.68 ± 1.29	0.005
Carcass composition (Moisture, %)^10^	77.93 ± 0.59	78.02 ± 0.09	0.807
Carcass composition (Protein, %)^11^	67.69 ± 1.52	67.27 ± 1.33	0.772
Carcass composition (lipid, %)^11^	14.31 ± 1.73	13.15 ± 1.84	0.394
Carcass composition (ash, %)^11^	15.55 ± 0.79	16.55 ± 0.89	0.183

Data are means ± SEM (n = 3).

^1^SGR = 100 * (Ln W_t_ − Ln W_0_)/t, where W_t_ and W_0_ represented the body weight of initial fish and final fish respectively, while *t* represented the rearing days of this experiment.

^2^WGR = (W_t_ − W_0_)/W_0_.

^3^FI = 100 × dry feed intake × 2/(W_t_ + W_0_) × t.

^4^HSI = 100* (liver weight/whole body weight).

^5^CF = 100*(body weight/body length^3^).

^6^FCR = (dry feed intake)/(W_t_ − W_0_).

^7^PER = (W_t_ − W_0_)/(dry feed intake*protein percent in dry diet).

^8^ADC, dry weight = (1−(Y% in diet)/(Y% in faeces))*100%.

^9^ADC, protein = (1−(Y % in diet)/(Y % in faeces)) * (% protein in faeces/% protein in diet)*(100%).

^10^Percentage of wet fish weight.

^11^Percentage of dry fish weight.

**Table 2:** Basal diet formulation.

**Ingredient**	**Percentage** (**%**)
Fish meal	65.00
Wheat meal	23.15
Soy lecithin	2.00
Fish oil	6.50
Vitamin premix^1^	0.50
Mineral premix^2^	1.00
Choline chloride	0.30
Attractants^3^	0.50
Calcium propionate	0.10
Ethoxy quinoline	0.05
Monocalcium phosphate	0.3
Y_2_O_3_	0.1
Binder (Na Alginate)	0.5
Proximate analysis	
Crude protein	50.09
Crude lipid	12.48

^1^Supplied the following (mg/kg diet): retinyl acetate, 32; cholecalciferol, 5; tocopheryl acetate, 240; menadione sodiumbisulphite, 10; ascorbic acid, 2000; cyanocobalamin, 10; biotin, 60; folie acid, 20; inositol, 800; niacin, 200; pantothenate, 60; pyridoxine HCL, 20; riboflavin, 45; thiamin HCL, 25; microcrystalline cellulose, 1473.

^2^Supplied the following (mg/kg diet):MgSO_4_·7H_2_O, 1200; CuSO_4_·7H_2_O, 10; FeSO_4_·7H_2_O, 80; ZnSO_4_·H_2_O, 50; MnSO_4_·H_2_O, 45; CoCl_2_,50; Na_2_SeO_3_, 20; calciumiodate, 60; Zeolite powder, 8485.

^3^Supplied the following (% dry diet): betaine, 0.4; DMPT, 0.2; threonine, 0.2; glycine, 0.1; inosine-5′-diphosphate trisodium salt, 0.1.

**Table 3:** Primers used in experiment.

**Genes**	**Sense primer**	**Reversed antisense primer**	**Amplification Size**	**Tm/ °C**	**E-value**
GK	CGACACGAGGACATTGACAAG	CCAACAATCATCCCGACTTCAC	218	60	1.063
PK	CCAGTTGCACCAGGATCAATA	CCAGTTGCACCAGGATCAATA	204	60	0.983
FAS	AGTGGTAGTGCTGCTGAC	CTATGTTTGCCTCCTGGTAG	164	60	0.993
Dgat2	TGCTGTGGTCATCGTTATC	CTTGTAGGCGTCGTTCTC	163	57.5	0.981
Soat2	GCTCGTGATGTTCGTCTAC	TGAATGGAGGACAAGATTAACC	129	57.5	0.993
SREBP1	GCCATTGACTACATCCGTTAC	CATCAGCCTGTCCATCTACTTC	136	60	0.993
cPEPCK	GTGTTTGTTGGAGCAGCCATGAG	GCTCTTGCGGAACCAGTTGACG	201	57.5	1.015
G6Pase	CACGAGACGGCTCATTATGC	CTTTGCTGCTGGATTTCTTGC	193	60	0.980
Cpt1a	ATGGGAAGAGTGGACTGAATG	GCTGGAAGGCATCTGTGG	96	57.5	0.996
Notch1	GACGGACCCAACAAATAC	GGTACAGATGTAGGCGTTCT	157	60	1.013
Hes1	GGAGAAGAGCCCGAATC	GGTCATCTGAGCCCTTTG	153	57.5	0.991
EF-1α	TATTAACATCGTGGTCATTGG	CAGGCGTACTTGAAGGAG	149	60	0.993
α-Tubulin	CCCTCGTATCCAITTCCCTC	GGTAGTTGATGCCCAICTTGA	208	60	0.993
